# Utilization of microalgae for agricultural runoff remediation and sustainable biofuel production through an integrated biorefinery approach

**DOI:** 10.1186/s40643-023-00720-w

**Published:** 2024-01-11

**Authors:** Qurrat ul ain Rana, Saira Latif, Saleha Perveen, Abdul Haq, Sidra Ali, Muhammad Irfan, Rahul Gauttam, Tawaf Ali Shah, Turki M. Dawoud, Gezahign Fentahun Wondmie, Mohammed Bourhia, Malik Badshah

**Affiliations:** 1 Sustainable Bioenergy and Biorefinery Laboratory, Department of Microbiology, Quaid-I-Azam University, Islamabad, 45320 Pakistan; 2 Present Address: Joint Genome Institute, Lawrence Berkeley National Laboratory, Berkeley, CA 94720 USA; 3 Present Address: Peshawar Laboratories Complex, Pakistan Council of Scientific & Industrial Research, Islamabad, 25120 Pakistan; 4 Department of Oral Biology, College of Dentistry, University of Florida, Gainesville, FL USA; 5 The Joint Bioenergy Institute, Emeryville, CA USA; 6 Collee of Agriculture Engineering and Food Sciences, Shandong University of Technology, Zibo, China; 7 Department of Botany and Microbiology, College of Science, King Saud University, P. O. BOX 2455, 11451 Riyadh, Saudi Arabia; 8 Department of Biology, College of Science, Bahir Dar University, P.O Box 79, Bahir Dar, Ethiopia; 9 Department of Chemistry and Biochemistry, Faculty of Medicine and Pharmacy, Ibn Zohr University, 70000 Laayoune, Morocco

**Keywords:** Chlorella, Biodiesel, Biogas, Lipase, Anaerobic digestion, *Bacillus subtilis*

## Abstract

Generally wastewater such agricultural runoff is considered a nuisance; however, it could be harnessed as a potential source of nutrients like nitrates and phosphates in integrated biorefinery context. In the current study, microalgae Chlorella sp. S5 was used for bioremediation of agricultural runoff and the leftover algal biomass was used as a potential source for production of biofuels in an integrated biorefinery context. The microalgae Chlorella sp. S5 was cultivated on Blue Green (BG 11) medium and a comprehensive optimization of different parameters including phosphates, nitrates, and pH was carried out to acquire maximum algal biomass enriched with high lipids content. Dry biomass was quantified using the solvent extraction technique, while the identification of nitrates and phosphates in agricultural runoff was carried out using commercial kits. The algal extracted lipids (oils) were employed in enzymatic trans-esterification for biodiesel production using whole-cell biomass of *Bacillus subtilis* Q4 MZ841642. The resultant fatty acid methyl esters (FAMEs) were analyzed using Fourier transform infrared (FTIR) spectroscopy and gas chromatography coupled with mass spectrometry (GC–MS). Subsequently, both the intact algal biomass and its lipid-depleted algal biomass were used for biogas production within a batch anaerobic digestion setup. Interestingly, Chlorella sp. S5 demonstrated a substantial reduction of 95% in nitrate and 91% in phosphate from agricultural runoff. The biodiesel derived from algal biomass exhibited a noteworthy total FAME content of 98.2%, meeting the quality standards set by American Society for Testing and Materials (ASTM) and European union (EU) standards. Furthermore, the biomethane yields obtained from whole biomass and lipid-depleted biomass were 330.34 NmL/g VS_added_ and 364.34 NmL/g VS_added_, respectively. In conclusion, the findings underscore the potent utility of Chlorella sp. S5 as a multi-faceted resource, proficiently employed in a sequential cascade for treating agricultural runoff, producing biodiesel, and generating biogas within the integrated biorefinery concept.

**Graphical Abstract:**

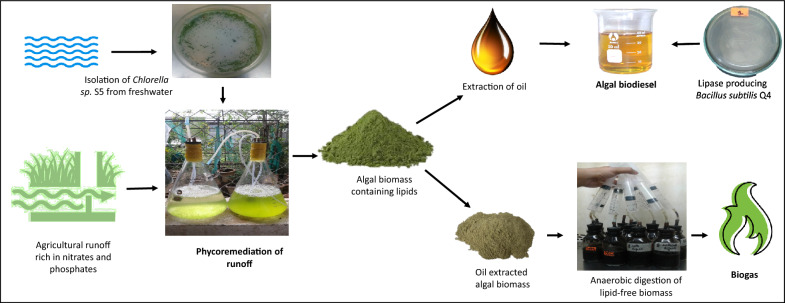

## Introduction

Water is one of the most important components of all living beings. Almost 70% of the total earth’s surface is covered by water, and only 3% of it is available for drinking as freshwater (Cao et al. 2019). Given the proven scarcity of water, its integrity should never be compromised. Annually around 3.4 million deaths and numerous water-borne disease outbreaks are reported due to water being polluted by industries, agricultural runoff, and wastewater treatment plants (Osiemo et al. 2019). Currently, agricultural runoff is the major contributor to freshwater pollution. The agricultural sector is solely dependent on a variety of fertilizers to enhance crop yield and their application has increased many folds in the last few decades. Nutrient concentration in water is increased when agricultural runoff containing phosphorous, and nitrogen falls into the water bodies. These are the key elements polluting the water reservoirs and causing surface water eutrophication (Hanrahan et al. 2019). However, this wastewater enriched in nitrates and phosphates is a perfect niche for the growth of microalgae. An imperative option is to treat agricultural runoff using microalgae. But the problem with the algal wastewater treatment process is the generation of algal biomass, which requires cost-effective management. Conversely, this algal biomass can be converted into biofuel by use of microbial valorization. In this way, wastewater treatment plants can serve as integrated biorefineries, which can also address the energy shortage faced by the world (Arora et al. 2021).

Microalgae are the ideal candidates to treat the agriculture runoff polluted with nitrates and phosphates. Previous studies have utilized microalgae to treat agricultural runoff, however they used only a single-step approach that ultimately lead to water pollution in the form of algal blooms (Shu et al. 2018). *Chlorella* is one of the most potent species of microalgae that can remove nutrients from agricultural runoff (Shu et al. 2018). Moreover, this species is capable of accumulating high amount of lipids as biomass that can further be exploited for biodiesel production (Chi et al. 2019). Other studies have targeted the algal biomass lipids for biodiesel production, leaving behind the algal lipid-depleted biomass as waste. Biodiesel is carbon neutral, environmental friendly renewable alternate of conventional petroleum diesel. There are two methods for biodiesel production: chemical and biological. Chemical methods utilize alkali or base as catalyst and require high reaction temperatures making them not only expensive but also generating alkali and acid-based wastewater, which is a hallmark for water pollution. Alternately, biological synthesis employs lipase-mediated trans-esterification at low temperatures that is a cost effective and environmental friendly method ( Rana 2019). The acquired biodiesel can be used in conventional engines without any further alterations (Rana et al. 2019). The leftover biomass remaining after the lipid extraction can be used to produce biogas, which is a mixture of methane and carbon dioxide generated through anaerobic digestion (Khan et al. 2017). To tackle these barriers, an integrated process is needed to treat wastewater using microalgae and convert this algal biomass into energy carriers. Transesterification and anaerobic digestion are the ideal and cost-effective technologies to, respectively, convert the algal lipids and residual algal biomass into biodiesel and biogas. This integrated approach will enhance the nutrient recovery from agricultural runoff, generate energy carriers, and mitigate water pollution. Thus, the phycoremediation of agricultural runoff followed by the treatment of algal lipids and the remaining algal biomass for biodiesel and biogas production through trans-esterification and anaerobic digestion, can serve an efficient and sustainable integrated biorefinery route to address the energy shortage and mitigate the water reservoir pollution. To the best of our understanding no such study has been reported on development of a lab-scale integrated wastewater biorefinery using microalgae.

In the present study, a sustainable approach was used to mitigate the agricultural runoff and subsequently employ the algal biomass for production of energy carriers in an integrated biorefinery context. Chlorella *sp.* isolated from freshwater was employed for bioremediation of agricultural runoff. The algal biomass was harvested after the removal of nutrients from wastewater and further utilized for lipid extraction. The extracted algal oil was used for biodiesel production using lipase-mediated trans-esterification using a whole cell-based approach. The lipid-depleted biomass and whole algal biomass were then used for production of biogas through anaerobic digestion process.

## Materials and methods

### Algal biomass

#### Sampling and isolation of microalgae

Microalgae were sampled from the freshwater stream at Quaid-i-Azam University, Islamabad, Pakistan. The sample was brought to the lab under sterile conditions and stored at 4 ^0^C. Physiochemical analysis of water sample was carried out and BG11 media was used to isolate and select pure algal isolates.

#### Identification of isolated microalgae

The isolated microalgae were identified by examining their cell size, shape, color, cell wall, and chloroplast shape using fluorescent microscope with a bright field microscopy.

#### Cultivation of isolated microalgae

Microalgae were cultivated on BG11 media by preparing three stock solutions. Stock solution 1 was prepared by dissolving 15 g NaNO_3_ in one liter of distilled water. Stock solution 2 was prepared by mixing 0.2 g of K_2_HPO_4_, 0.375 g of MgSO_4_, 0.18 g of Ca (NO_3_)_2_, 0.03 g ml of citric acid, 0.03 g of FeCl_3_, 0.005 g of EDTA and 0.1 g of Na_2_CO_3_ per 50 mL of distilled water. Stock solution 3 was composed of micronutrients; 0.03 g H_3_BO_3_ 0.02 g MnCl_2_.4H_2_O, 0.002 g, ZnSO_4_.7H_2_O, 0.004 g Na_2_MoO_4_.2H_2_O, 0.001 g CuSO_4_.5H_2_O and 0.0005 g CoCl_2_ per 10 mL of distilled water. 1 L BG11 media was prepared by mixing 100 mL, 10 mL, and 1 mL of stock solutions 1, 2, and 3, respectively. As microalgae are photosynthetic; artificial aeration using aeration pumps and fluorescent light were provided for algal cultivation. To produce biofuels, a large amount of biomass was produced by transferring 50 mL of algal culture to 5000 mL of media in a 5L flask and incubation was carried out in natural daylight and temperature in order to mimic the conditions present at wastewater treatment plants.

### Growth optimization of isolated microalgae

To achieve excessive algal biomass with a high concentration of accumulated lipids, growth conditions were optimized by varying nitrates, phosphates, and pH. Normal concentrations of nitrate and phosphate for BG11 media are 1.5 g and 0.0.6 g per liter of distilled water, respectively. To identify suitable concentrations for maximum growth five different concentrations of NaNO_3_ (g. L^−1^; 0.75, 0.125, 1.5, 1.875, 2.25) and K_2_HPO_4_ (g. L^−1^; 0.02, 0.04, 0.06, 0.08, and 0.10) were used. The optimum growth conditions of algae were checked under continuous illumination and aeration.

Similarly, five different pH ranges (6.5, 7, 7.5, 8, 8.5) were used to find out the maximum growth. The growth was measured through optical density at 680 nm by using a spectrophotometer.

### Growth kinetics and lipid content determination

To asses growth kinetics and determine lipid contents, dry biomass acquired from the exponential growth phase was measured using biomass productivity (Pdwt), expressed in grams per liter per day. Cells were separated by centrifugation for 5 min at 4 °C and a speed of 4,000 RPM in order to determine Pdwt biomass. Following centrifugation, pellet was dried in an oven, rinsed with distilled water, and weighed using an analytical scale. The specific growth rate of the algae was determined by measuring variation between the number of cells at the initial and final logarithmic phases.

For biodiesel production lipid is an important substrate, therefore determination of the microalgal capability of lipid production was an important step. Total lipids content (Lc) is the percent of lipids in total biomass (% dwt), determined by using the n-hexane method. Centrifugation was used to obtain microalgal biomass as described earlier. The supernatant was removed, and the pallet was dried in an oven at 105 ˚C for 3 h. Biomass was then milled to powder and weighed. To powdered biomass, n-hexane and isopropanol (3:2) were added to a flask and agitated at 210 rpm for 19 h. Filtration was then carried out to remove solid residues (Bharathiraja et al. 2014). All the algal lipids were extracted in the filtrate. Filtrate was then transferred to a separatory funnel and more n-hexane and distilled water was added to separate lipids from other soluble compounds. The biphasic layer then appeared with the lower aqueous layer containing water-soluble compounds and the upper n-hexane layer, which contained soluble lipids. n-hexane was separated from lipids by rotary (Joseph 2017). The formulae used for the determination of biomass productivity, specific growth rate, volumetric lipid yield, and lipid content are provided in the supplementary data file.

### Treatment of agricultural runoff using microalgae

Agricultural runoff was generated in the lab by curing 500 g of soil obtained from a freshly fertilized cornfield in 5 L of water for 1 week. After one week water was filtered and used as agricultural runoff. Using a Total Nitrogen Determination Kit and Phosphate Determination Kit from Merk, NY, USA, nitrates and phosphates in agricultural runoff were identified. The analysis was carried out using Spectroquant® Prove UV/VIS spectrophotometer (Merck, NY, USA). About 10 ml of algae were inoculated in 1 L agricultural runoff. Nitrate and phosphate analysis was performed to observe the nutrient removal potential of selected microalgae strains after regular intervals (APHA, 21st Edition).

### Lipase mediated trans-esterification of algal oil

For the biological trans-esterification of oil, lipase-producing bacteria were used through a whole-cell approach. Bacterial strain Q4 was isolated from vegetable oil industry wastewater and screened for lipase production in a previous study and was utilized for this purpose (Rana 2019; Haq et al. 2020).

### Culture enrichment

Culture enrichment was carried out in nutrient broth. About 20 mL nutrient broth in 100-ml Erlenmeyer flask was inoculated with two loops full of culture under sterile conditions. The flask was kept in a shaker for 24 h at 37˚C and 150 RPM.

### Identification of lipolytic bacterial strain Q4

Both physiological and genetic identification processes were used to identify Q4. Q4 was biochemically identified by the standard protocol in the literature (Whitman et al. 2012). For molecular identification, the DNA of bacteria was extracted first via the cetyltrimethylammonium bromide method described by Wilson (Wilson 2001). The collected DNA was then subjected to commercial 16S ribosomal RNA sequencing for molecular identification of Q4 using Macrogen Standard Custom DNA Sequencing Services (Macrogen Inc., Seoul, Korea).

### Lipase production through submerged fermentation and lipase assay

From 24-h enriched bacterial culture 5% was inoculated in 250 Erlenmeyer flasks having 50 ml enzyme production media (*w*/*v*: peptone 0.2%, NaCl 0.25%, KH_2_PO_4_ 0.1%, CaCl_2_, 2H_2_O 0.04%, MgSO_4_.7H_2_O 0.04%, 1–2 drops of Tween-20 and olive oil 2%). The flask was then incubated in a shaker at 150 RPM and 37 °C for 24, 48, and 72 h. After incubation, 5 ml of the media was taken out and centrifuged at 4000 rpm for 10 min. The supernatant was stored at – 20 ºC while the pellet was discarded. The supernatant was used to determine enzyme activity. Lipase activity was measured as previously described by Kumar et al. (Kumar et al. 2005) with some modifications. About 20 Mm of p-nitrophenyl laurate solution was prepared in isopropanol and acetonitrile in a 1:1 ratio. About 75 µl of this substrate solution was added to 25 µl of enzyme solution (supernatant). To make the final volume of solution 3 ml, Tris HCl buffer at pH 7 was added. The reaction solution was then incubated at 37 ºC for 30 min. After incubation enzyme activity was stopped by a quick reduction of solution temperature to freezing level. The precipitates from the chilled solution were removed by centrifugation at 10000 rpm for 5 min. The absorbance of released PNP was measured at 410 nm. A standard curve was used to determine the unknown concentration of released PNP. The heat-inactivated enzyme was used as the negative control's "blank control". Absorbance is measured at 410 nm for making the standard curve for PNP from the 10–100 M solution of PNP in a Tris–HCl buffer at a pH 7. An enzyme's potential to catalyze the release of 1 µM para-nitrophenol every minute each ml under standard assay conditions (temperature 37 °C, pH 7), was defined as one unit of enzyme activity.

### Methanol toxicity test

Strain Q4 was checked for its methanol sensitivity as methanol was used for biodiesel production and it is toxic to bacteria. In autoclaved nutrient broth methanol was added in 5%, 10%, and 15% concentrations which were equivalent to the concentrations of methanol used for the trans-esterification reaction (Heipieper et al. 1994). Almost 5% of 24 h fresh nutrient broth enriched culture was inoculated in nutrient broth containing methanol at different concentrations and incubated at 37 °C and 150 RPM. Growth OD was analyzed at a wavelength of 680 nm after 24, 48, and 72 h of incubation.

### Bacterial mediated trans-esterification of algal oil

Biodiesel was produced via lipolytic bacterial strain Q4 from lipids biomass of microalgae using a whole cell-based approach. Cell mass was generated by growing the strain in LB broth (peptone 10 g/L, NaCl 5 g/L, and yeast extract 5 g/L) (Rana 2019). The bacterial culture (5%) was incubated in 100 mL of LB broth at 37 ºC and 150 RPM for 48 h to achieve maximum biomass. After 48 h, the reaction mixture was centrifuged at 4000 RPM for 15 min; followed by supernatant removal, and the pallet was stored at 4 ºC in normal saline. About 1 mL of pelleted cells was added to 1:6 ratios of oil and methanol. The added cells were mixed initially in the oil layer to avoid any inhibition by direct exposure to methanol. Inoculum was used in bulk to ensure cell activity if some of the cells get inhibited by methanol. Methanol was added afterward, and the mouth of the flask was tightly closed to avoid any evaporation. About 300 µL of n-hexane was added as solvent and emulsifier to increase the surface contact area of oil and methanol, which ease the lipase access to components for trans-esterification reaction (Devanesan et al. 2007). The reaction mixture was then incubated at 37 °C and 150 RPM for 48 h. After 48 h, flask’s contents were transferred to static test tubes for 24 h to separate layers. After complete settling and layer formation, the upper yellow layer of biodiesel was collected by using a micropipette.

### Analysis

Fatty acid methyl esters (FAMEs) formed after trans-esterification was analyzed using Fourier transform infrared spectroscopy (FTIR) and gas chromatography–mass spectrometry (GC–MS) chromatogram. Methyl ester formed after trans-esterification was analyzed by FTIR spectrophotometer of Bruker, model tensor 27 with software version OPUS 65, equipped with ZnSe ART. 5 µl sample of biodiesel was loaded at the sample injector. A total of 16 scans were performed in a range of 400–4000 cm^−1^ and the average was represented in the form of a spectrum with different peaks ranges. The GC–MS was performed to assess the quality and quantity of biodiesel FAME contents. Capillary column (DB-5MS Agilent; 30 m × 0.25 mm; 0.25 mm film thickness) using helium at a flow rate of 1.5 ml/min as carrier gas was used. The rise in column temperature was programmed at 50–300 °C at a rate of 10 °C/min. The temperature of both detector and injector was kept at 250 °C and the mass spectrometer was set to scan with ionization in electron impact mode in the range of m/z 50–550. In order to quantify the amount of FAMEs in biodiesel, methyl acetate was used as an internal standard.

### Anaerobic digestion of oil-free algal biomass for production of biogas

Two types of substrates were used for biogas production including lipid extracted algal biomass and completely algal biomass (lipids extracted).

### Determination of total and volatile solids

The effluent slurry from running an experimental biogas reactor in the Sustainable Biorefinery and Bioenergy Lab of Quaid-i-Azam University Islamabad was used as inoculum. Total solids (TS) and volatile solids (VS) determination of both inoculum and substrates was carried out as described previously (Sluiter et al. 2008).

### Experimental setup for batch process

In batch setup, 100-mL glass reactors with 70 mL working volume were used for anaerobic digestion. They were connected to vials (60 mL) for the collection of biogases. The inoculum-to-substrate ratio was kept at 4:1. Inoculum was used as a negative control to determine biogas production in the background. Starch was used as a positive control as a reference to confirm inoculum activity (Holliger et al. 2016). The calculated amount of substrate and inoculum was added to each reactor, and the reactors were purged with nitrogen gas to remove air. The reactors were then air-tightened with butyl rubber corks having outlets for gas collection and incubated at 37 °C for 50 days. Biogas production was recorded routinely until reactors reached a steady state where the rate of biogas production became constant. A scrubbing solution (3 M NaOH) was used to remove CO_2_ and release CH_4_ (Abdeen et al. 2016). The accumulated biogas and methane yields were converted to normalized values at standard temperature and pressure (Table 1).

**Table 1:** Composition of inoculum and substrate in batch experiment

S.no.	Parameters	Conc
1	Total nitrogen	55 mg/L
2	Nitrate	5.3 mg/L
3	Total dissolved solids	48 mg/L
4	pH	7.6
5	Electrical conductivity	74 dsm^−1^
6	Salinity	78 µ cm
7	Total solids	134 mg/L
8	Sulfates	25 mg/L
9	Phosphate	43 mg/L

## Results

### Algal biomass production

In the first phase of the study, microalgae were isolated, identified, and cultivated to generate biomass to produce biofuels specifically biodiesel and biogas. For cultivation, two methods were employed: optimization on BG11 media and agricultural runoff.

### Physicochemical characterization

The physicochemical analysis of the freshwater, from which microalgae strain S5 was obtained, was conducted to identify and estimate the growth requirements of the microalgae (Table 2).

**Table 2:** Physicochemical characterization of fresh water from which microalgae was isolated

S.no.	Parameters	Conc.
1	Total nitrogen	55 mg/L
2	Nitrate	5.3 mg/L
3	Total dissolved solids	48 mg/L
4	pH	7.6
5	Electrical conductivity	74 dsm^−1^
6	Salinity	78 µ cm

### Identification of isolated microalgae

**Fig. 1:**
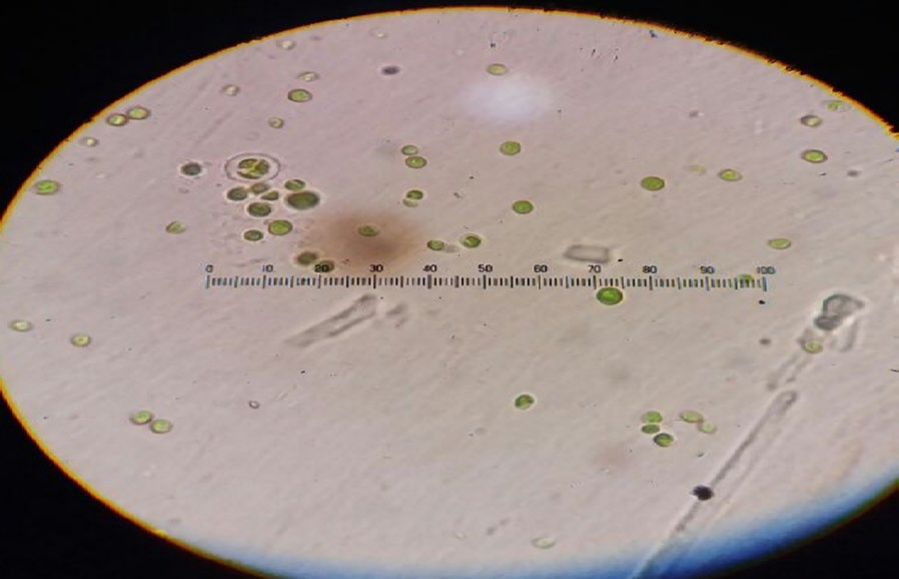
Light microscopy image of Chlorella sp. strain S5

The morphological analysis of strain S5 using bright field light microscopy at 40X magnification revealed that the microalgae were unicellular, and cells were non-motile, solitary, and spherical having a diameter of about 2–4 µm. The cells had prominent central rounded chloroplasts and the cell wall was a single smooth layer **(**Fig. 1**).**

### Growth optimization of chlorella sp. S5

**Fig. 2:**
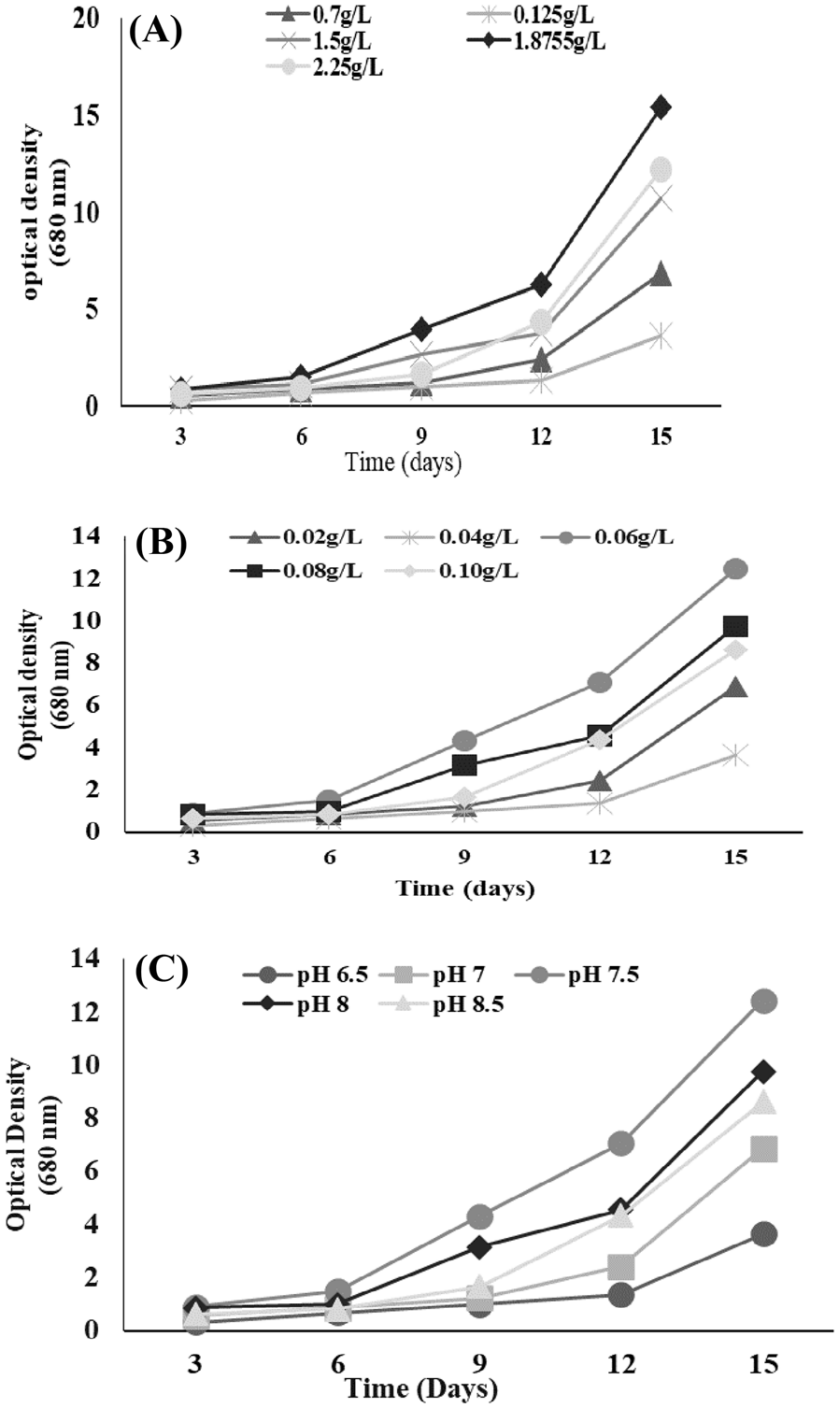
Growth optimization of microalgae at different concentration of **A** NaNO_3_ **B** K_2_HPO_4_ and **C** pH

Growth optimization of the Chlorella sp. S5 was carried out to enhance its biomass in order to achieve a higher yield of targeted biofuels. For this purpose, BG11 media previously used for algal growth was optimized in terms of nitrates, phosphates, and pH. The normal concentration of NaNO_3_ in BG11 media is 1.5 g/L while the concentration of K_2_HPO_4_ is 0.06 g/L. BG11 media was modified by changing the concentration of nitrates and phosphate to know at which concentration of nitrate and phosphate the selected strain shows maximum growth. Five different concentrations of NaNO_3_ (g/L: 0.75, 0.125, 1.5, 1.875, 2.25) and five different concentrations of K_2_HPO_4_ (g/L: 0.02, 0.04, 0.06, 0.08, 0.10) were used. Similarly, five different pH ranges (6.5, 7, 7.5, 8, 8.5, 9) were used, as the pH of the original media was 7. Chlorella sp. strain S5 showed the highest growth when NaNO_3_ concentration was 1.875 g/L, K_2_HPO_4_ concentration was 0.06 g/L, and at pH 7.5 (Fig. 2A, B and C).

### Effect of seasonal variation on biomass production and lipid accumulation

The current study was focused on developing a wastewater treatment plant that employed algae as a phytoremediation for agricultural runoff and using the generated algal biomass for biodiesel and biogas production. Thus, microalgae were grown in a 5-L flask under natural temperature and daylight conditions and the effect of seasonal variation on the production of algal biomass and accumulated lipids were observed. Biomass production was mainly affected by temperature. Low temperature retarded the growth of microalgae, whereas an increase in temperature increased biomass production. However, growth was not inhibited even in extreme winter when the average lowest temperature became 4 °C and in extreme summer when the average highest temperature became 39 °C. In winter, the growth of strain S5 was 1.53 g/L while in the spring season, the growth was maximum up to 1.7 g/L. However, interestingly when we look at lipid accumulation, a higher amount of lipids was accumulated during the winter season (Table 3).

**Table 3:** Characteristics of Chlorella sp*.* S5 biomass when grown in different seasons

Months	Biomass (dry wt.) g/L	Lipid content % dry wt	Growth time (days)	Biomass productivity (g.L^−1^ day^−1^)	Volumetric lipid productivity (mg.L^−1^ day^−1^)
December	1.52	16	30	0.045	24
January	1.6	18	30	0.048	28.8
February	1.37	15	30	0.041	20.5
March	1.37	17	30	0.041	23.2
April	1.7	17	30	0.052	29.4
May	1.73	16	30	0.052	27.7

### Growth kinetics and lipid content determination

Growth kinetics and lipid content determination were conducted in order to evaluate the potential of Chlorella sp. S5 for biofuels mainly biodiesel and biogas production. For this purpose, microalgae were cultivated at optimized parameters and ambient temperature for 26 days. The specific growth rate of S5 was found to be 0.14µ/day, biomass productivity was 0.29 gL^−1^ day^−1^ and lipid content was found to be 16% while the volumetric lipid productivity was 46.4 mgL^−1^ day^−1^ (Table 4).

**Table 4:** Growth rate, lipid content and volumetric lipid productivity of Chlorella sp*.* S5

Max. absorbance	7.99881
Min. absorbance	0.1296
Days	26
Specific growth rate (µ = lnX-lnX◦/No of days)	0.14
Biomass productivity Pdwt (gL^−1^ day^−1^)	0.29
Lipid content (LC; % dwt)	16%
Volumetric lipid productivity (mgL-1 day-1)	46.4

### Treatment of agricultural runoff using *Chlorella sp.* S5

**Fig. 3:**
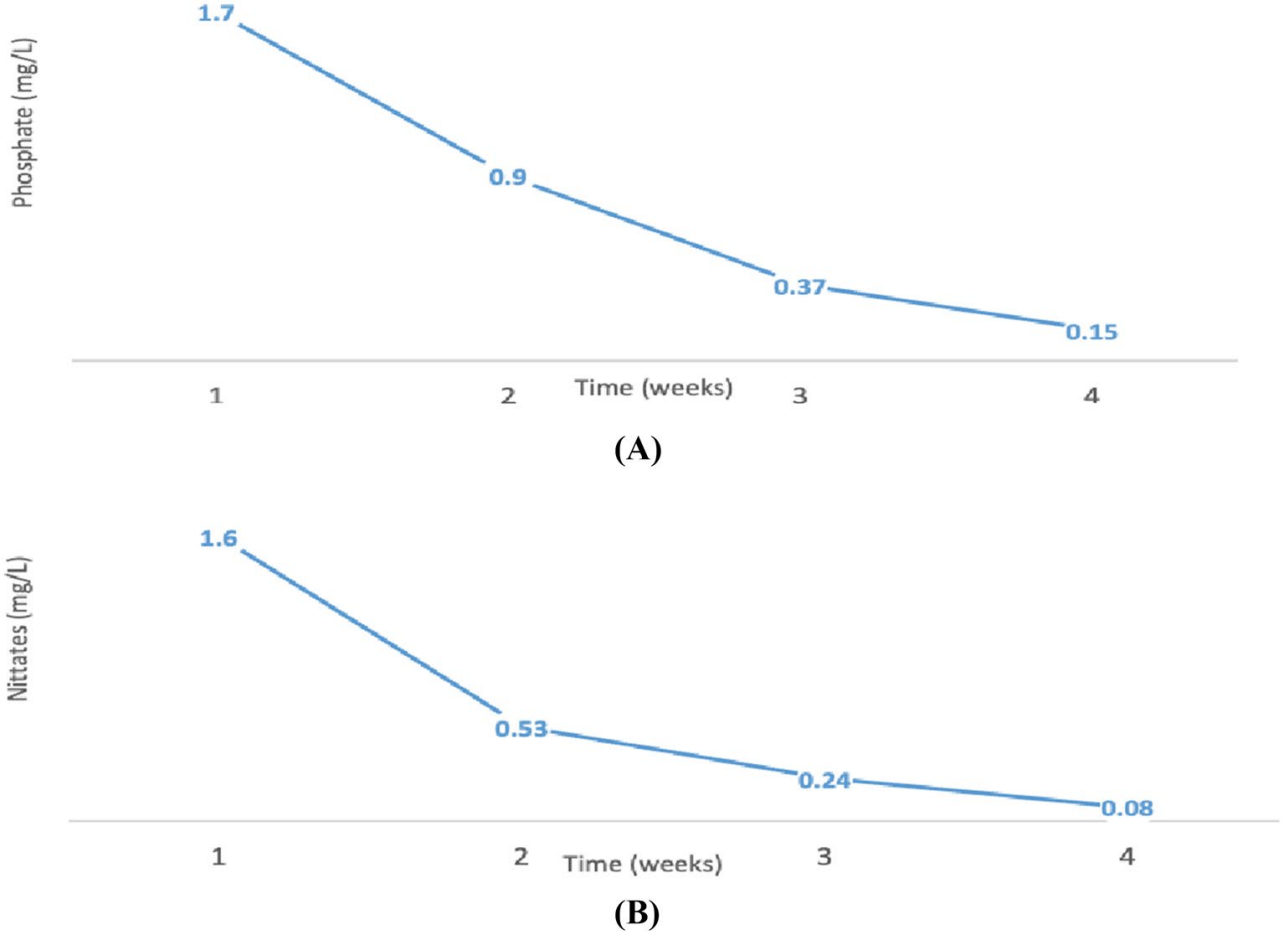
Reduction of **A** phosphates, **B** nitrates from agricultural runoff using Chlorella sp*.* S5

Agricultural runoff contains enough amounts of nitrates and phosphates that can sustain good growth of algae. For this purpose, S5 was cultivated in agricultural runoff having an initial nitrate concentration of 1.6 mg/ml and an initial phosphate concentration of 1.7 mg/ml. The wastewater was treated with algae for 4 weeks and about 91% reduction in phosphate and 95% reduction in nitrates were achieved (Fig. 3a, b). While cultivating algae in agricultural runoff, *Chlorella sp.* S5 was also cultivated in optimized BG11 media at the same time and conditions in order to compare the biomass and lipid productivity of algae grown in wastewater and chemical media (Table 5).

**Table 5:** Comparative analysis of biomass and lipid productivity of Chlorella sp*.* S5 biomass cultivated from agricultural runoff and optimized BG11 media

*Chlorella* sp. S5 biomass	Lipid content % dry wt	Growth time (days)	Biomass productivity (gL-1.day-1)	Volumetric lipid productivity (mg.L-1 day-1)
Agricultural runoff	15.8	28	0.26	41
Optimized BG11 media	16	28	0.28	44

### Lipase mediated trans-esterification of chlorella sp. S5 Oil

Chlorella sp. S5 depicted the potential of accumulating high lipid content in its biomass so in the second phase of the study its oil was subjected to bacterial valorization using trans-esterification to produce biodiesel. The lipid-extracted algal biomass was saved for anaerobic digestion.

### Identification of lipase-producing bacterial strain Q4

**Fig. 4:**
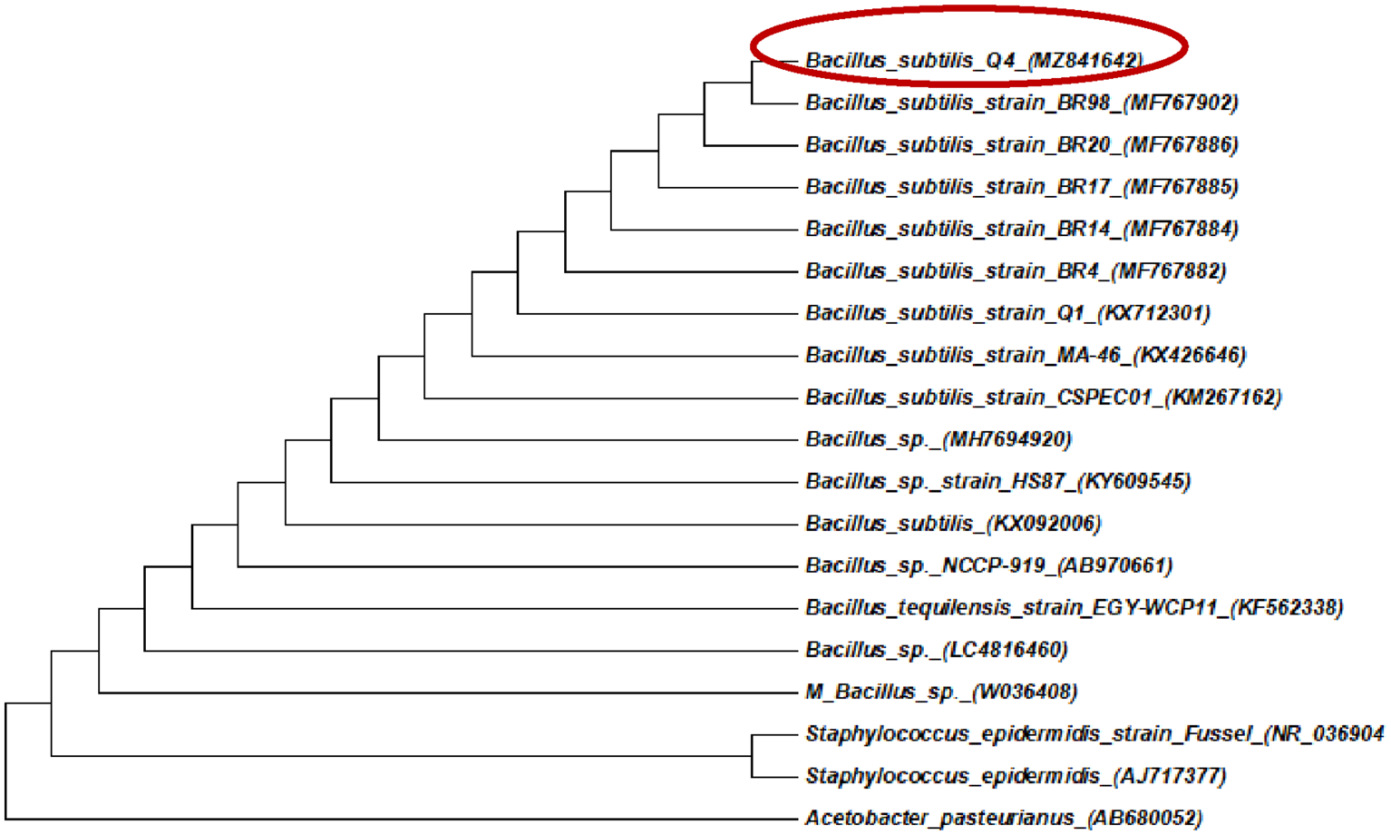
Phylogenetic tree for partial 16S rRNA gene sequences from bacterial isolate Q4 showing relationships between presented strains and related sequences

Lipase-producing bacterial strain Q4 previously isolated from vegetable oil industry wastewater was used to produce biodiesel from algal oil (Veerapagu et al. 2016). Strain Q4 was biochemically and molecularly identified. The biochemical characterization of strain Q4 showed that it belonged to *Bacillus* sp*.* (Table 6). The phylogenetic tree was constructed from the 16S RNA sequence of strain Q4 using the neighbor-joining method (Fig. 4). The results of sequence analysis showed that strain Q4 was *Bacillus subtilis.* Further, the sequence of strain Q4 was submitted to GenBank, and its accession number was acquired. Strain Q4 was thus designated as *B. subtilis* Q4 MZ841642.

**Table 6:** Biochemical identification of bacterial strain Q4

Isolate	Catalase	Nitrate	Citrate utilization	TSI	Urease	Oxidase	Methyl-Red	VP	Identification
Q4	+	+	+	A/A	–	+	–	+	*Bacillus sp.*

### Lipase assay

**Fig. 5:**
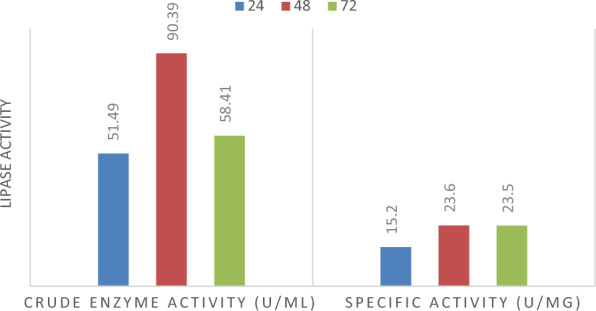
Crude and specific lipase activity of *B. subtilis* Q4 MZ841642 at 24, 48 and 72 h

Lipase assay was conducted to evaluate both crude and specific enzyme activity of *B. subtilis* Q4 for 24, 48, and 72 h. Crude Enzyme Activity of lipase produced by strain Q4 was highest after 48 h. While significant activity was observed after 24 h and slightly reduced after 72 h. Specific enzyme activity was highest for both 48 and 72 h of incubation while significant activity was recorded after 24 h of incubation (Fig. 5).

### Methanol toxicity

**Fig. 6:**
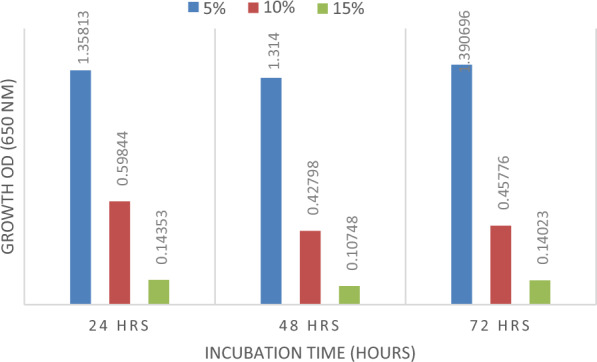
Growth of *B. subtilis* Q4 at varying methanol concentrations

During trans-esterification or alcoholysis, the alcohol reacts with oils producing FAMES and glycerol (Yingying et al. 2012). The most employed alcohols for this purpose are ethanol and methanol. In this study, methanol was used for trans-esterification. However, methanol is inhibitory to microorganisms as well as enzymes. *B. subtilis* Q4 was tested for methanol sensitivity by inoculating it into nutrient broth containing levels of methanol (5, 10, and 15%) corresponding to the concentration of methanol used for actual trans-esterification reaction, and incubating for 24, 48, and 72 h. The growth of bacteria was monitored using optical density measurements. Strain Q4 showed abundant growth at 5% methanol at 24, 48, and 72 h. Similarly, it also showed efficient growth at 10% methanol at all the incubation points. However, low growth was observed at 15% methanol concentration at 24, 48, and 72 h at 37 °C (Fig. 6).

### *B. subtilis* Q4 MZ841642 mediated trans-esterification of Chlorella sp. S5 oil

**Fig. 7:**
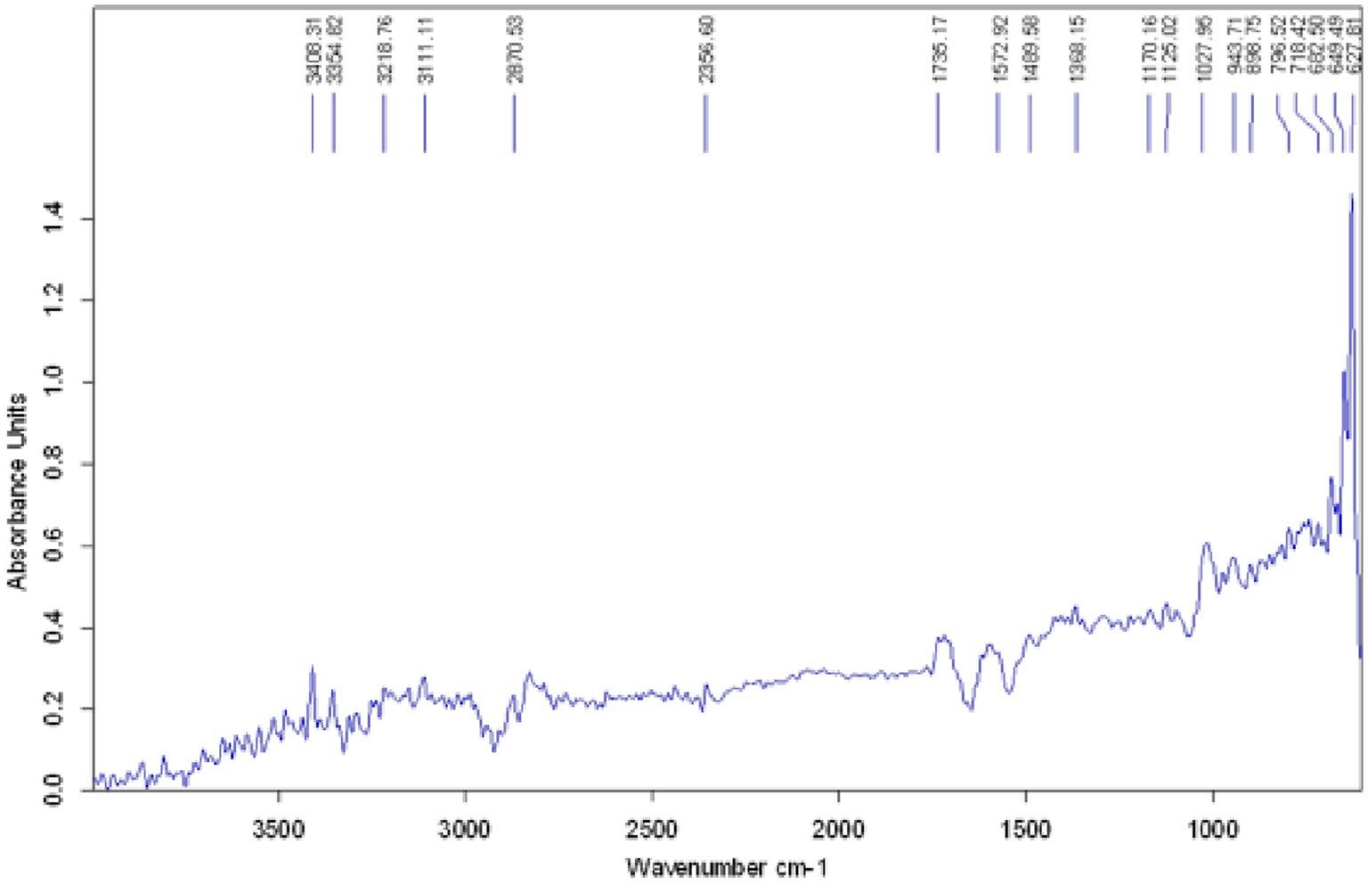
Infrared spectrum of biodiesel produced by *B. subtilis* Q4 mediated trans-esterification of Chlorella sp. S5 oil

Biodiesel was produced via a trans-esterification process using *B. subtilis* Q4 with algal oil. The production of biodiesel production from algal oil was confirmed by running FTIR. The infrared spectrum of biodiesel usually contains peaks in the frequency (cm^−1^) range of 1735–1750. The FTIR analysis showed a peak at 1735.17 cm^−1^, which confirmed the production of biodiesel (Fig. 7).

### Quality assessment and characterization of algal biodiesel

The quantity and quality of the FAMEs were assessed using GC–MS. The GC–MS chromatogram showed that the total FAME content of biodiesel produced was 98.2%. The FAME content of algal biodiesel was methyl palmitate (32.1%), methyl heptadecanoate (11.2%), methyl myristate (8.2%), methyl lineolate (29.8%), methyl stearate (6.2%), methyl 10-heptadecanoate (7.7%) and methyl hexanoate (3%).

### Anaerobic digestion of oil-free chlorella sp. S5 to produce biogas

In the third phase of the study, lipid-extracted algal biomass was anaerobically digested to produce biomass. In this way, the study was successful in developing an integrated wastewater biorefinery process.

### Determination of total solids and volatile solids of algal biomass and inoculum

The algal lipids and leftover dried mass were used as biomass for anaerobic digestion. TS and VS of both these substrates were determined to calculate the inoculum-to-substrate ratio in terms of weight to be used within the reactor (Table 7).

**Table 7:** Total solids and volatile solids content of substrates used for anaerobic digestion

Substrate	TS (%)	VS of TS (%)	Moisture (%)	VS of sample (%)
Inoculum	4.2%	72%	95%	3%
Strain S5 whole biomass	90%	88.89%	10%	88%
Lipid extracted biomass	88%	81.82%	12%	72%

### Anaerobic digestion

**Fig. 8:**
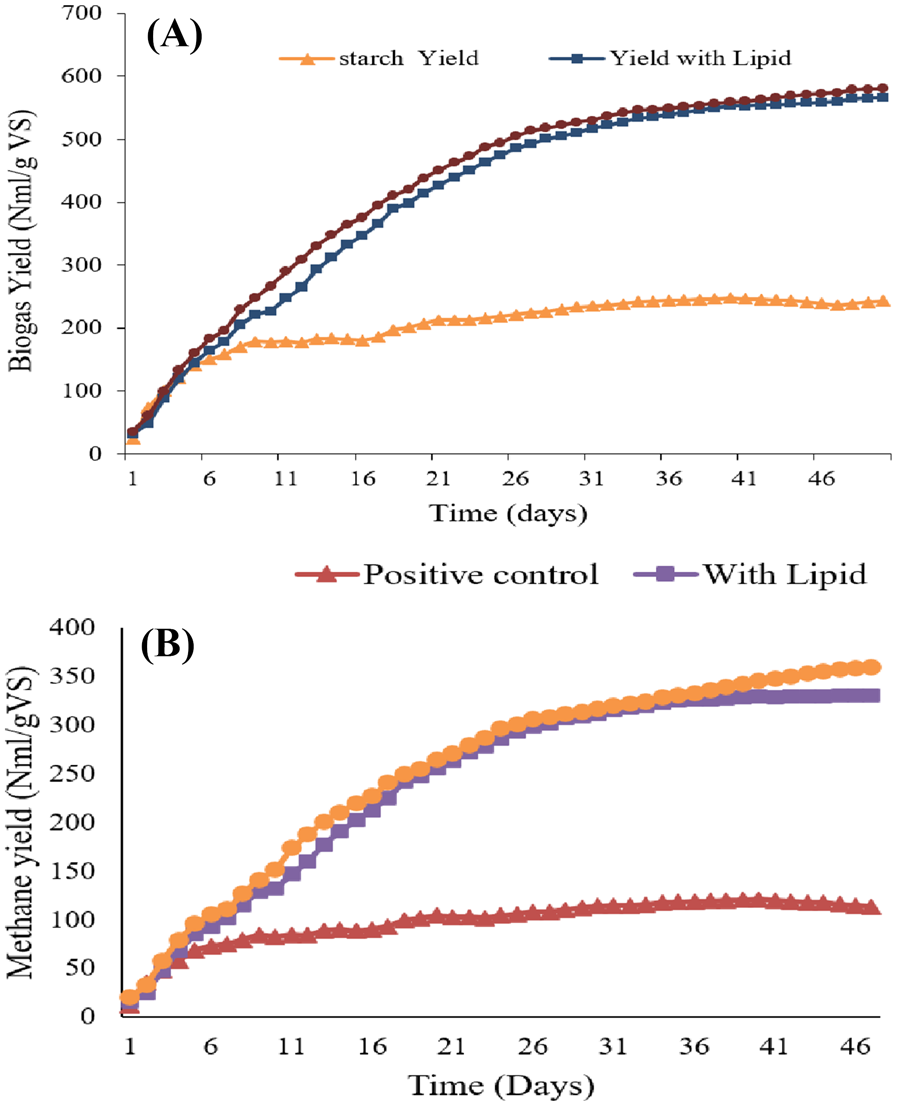
**A** Biogas yield using Chlorella sp. S5. **B** Biomethane yield using Chlorella sp. S5

The substrates including lipids, lipid-extracted biomass, starch (positive control), and inoculum (negative control) were used in batch anaerobic digestion mode for 50 days. Figure 8A and B shows that biogas and biomethane yield obtained from lipid-extracted biomass was 580 NmL/g VS_added_ and 364.34 NmL/g VS, respectively. Biogas and biomethane yield obtained from whole biomass were 580 NmL/g VS_added_ and 330.34 NmL/g VS_added_, respectively.

## Discussion

During the current study, microalgae were isolated, identified, and cultivated to produce biomass to produce biodiesel and biogas. Optimized BG11 media and agricultural runoff were used in this study. This is important to compare the biomass and lipid productivity and agricultural runoff was selected to generate biomass. During this study, utilization of wastewater had provided two advantages: such as bioremediation and cost-effective biomass production which was used as input for biodiesel production. The current finding agreed with the reports of Amaro et al. (Amaro et al. 2023) who reported that microalgae were important in wastewater treatment along with biomass production.

As morphology of microalgae and the physicochemical characteristics of freshwater exhibited that microalgal species was typical belongs to the genus Chlorella*.* Hence, the isolated microalgae strain was designated as Chlorella sp. strain S5. Chlorella species is known to produce and accumulate lipids in its biomass, which makes it an appropriate candidate to be cultivated for biodiesel production (Mata et al. 2010; Minhas et al. 2016).

For the growth of microalgae, the presence of key nutrient is important to get the desired amount of biomass. During this study, Chlorella sp. strain S5 showed the highest growth when NaNO_3_ concentration was 1.875 g/L, K_2_HPO_4_ concentration was 0.06 g/L, and at pH 7.5. Other study also indicated that, nitrates and phosphates are the main constituents required for its growth and they impact both growth of algal biomass and lipid production (Li et al. 2019). Similarly, pH plays a vital role in regulating the growth of microalgae. Most of the freshwater algae species grow at an alkaline pH range of 7.8–10 (Cassini et al. 2017).

In this study, microalgae were grown in a greenhouse under natural temperature and daylight conditions and the effect of seasonal variation on the production of algal biomass and accumulated lipids were observed. Biomass production was mainly affected by temperature. Especially, low temperature may retard the growth of microalgae, whereas an increase in temperature may also increase biomass production. However, in this study, the growth was not inhibited even in extreme winter when the average lowest temperature became 4 °C and in extreme summer when the average highest temperature became 39 °C. In the current study, the growth of strain S5 was 1.53 g/L during winter while the growth was 1.7 g/L in the spring season. However, interestingly when we look at lipid accumulation, a higher amount of lipids was accumulated during the winter season. This lipid accumulation might be due to low-temperature stress that microalgae suffered during the winter season. Various studies have also reported that, the application of low temperatures could enhance lipid accumulation in microalgae (Aratboni et al. 2019; Tan et al. 2018; Yuan et al. 2019). The current results showed that *Chlorella* sp. S5 can easily be used in wastewater treatment plants without requiring temperature controls hence, making the wastewater plant more feasible economically.

The growth kinetics and lipid content determination of Chlorella sp. S5 was conducted to evaluate its potential for biofuels (biodiesel and biogas) production. The specific growth rate of *Chlorella* sp. S5 was found to be 0.14 µ/day, biomass productivity was 0.29 gL^−1^ day^−1^ and lipid content was 16% while the volumetric lipid productivity was 46.4 mgL^−1^ day^−1^. These results indicated that there was a high biomass and oil productivity rate, and this showed that Chlorella sp. S5 is an excellent candidate to produce a high yield of biodiesel from algal oil and a high yield of biogas from lipid-extracted algal biomass if used to produce biofuels in a biorefinery approach. Similarly, the reports of Dahiya et al. (Dahiya et al. 2021) showed that high biomass productivity and high lipid content was produced by Chlorella sp.

During this study, agricultural runoff was also confirmed that it contains abundant amounts of nitrates and phosphates for the growth of algae. These excessive nutrients in agricultural runoff are responsible for algal blooming and water pollution (Bailey et al. 2020; Wurtsbaugh et al. 2019). However, this ability of algae to sequester nitrates and phosphates can be used for the treatment of agricultural runoff and afterward using the biomass to produce biofuels. In this way, wastewater plants can be converted into biorefineries for biofuel production. For this purpose, Chlorella sp*.* S5 was cultivated in agricultural runoff having an initial nitrate concentration of 1.6 mg/ml and an initial phosphate concentration of 1.7 mg/ml. The wastewater was treated with algae for 4 weeks and about 91% reduction in phosphate and 95% reduction in nitrates were achieved. The results showed the excellent ability of Chlorella sp. S5 for bioremediation of agricultural runoff. Similarly, Castellanos-Estupiñan et al*.* (Castellanos-Estupiñan et al. 2022) reported that Chlorella sp. has the highest removal efficiency of agricultural runoff such as NO_3_, PO_4_ and pesticides.

While cultivating algae in agricultural runoff, *Chlorella sp.* S5 was also cultivated in optimized BG11 media at the same time and conditions to compare the biomass and lipid productivity of algae grown in wastewater and chemical media. The results revealed that the biomass and lipid productivity of S5 remains similar in both cultivation conditions. It could be because the nitrates and pH conditions of wastewater were very similar to that of optimized conditions. Moreover, wastewater may also contain some additional nutrients, which further assisted algal growth. Hence, the biomass generated after wastewater treatment comprises sufficient oil and oil-extracted biomass to be used for biodiesel and biogas production. Thus, the growth of algae in wastewater not only cuts down the cost of chemical media thus making biofuel production a cheaper process, but also provides the advantage of agricultural runoff bioremediation (Castellanos-Estupiñan et al. 2022).

The biochemical characterization of strain Q4 and the phylogenetic tree that was constructed from the 16S RNA sequence of strain Q4 using the neighbor-joining method showed that strain Q4 was *Bacillus subtilis.* Indeed, the sequence of strain Q4 was submitted to GenBank, and its accession number was acquired, and it was designated as *B. subtilis* Q4 MZ841642. This bacterial strain has ability to produce lipase which was played a high role in the production of biodiesel /FAME/. Other study also reported that *Bacillus subtilis* species was one of promising lipase-producing bacteria (Mazhar et al. 2017).

Crude enzyme activity of lipase produced by *B. subtilis* strain Q4 was highest after 48 h. While significant activity was observed after 72 h. Specific enzyme activity was highest for both 48 and 72 h of incubation while significant activity was recorded after 24 and slightly reduced after hours of incubation. *B. subtilis* strain Q4 exhibited a high potential for lipolytic activity, which implies its tendency for biodiesel production. *Bacillus* species are known for the production solvent-stable lipases as it was reported by Gupta et al*.* (Gupta and Khare 2009).

In current study, methanol was used for trans-esterification even though it is inhibitory to microbes and enzymes. Lipases in particular are sensitive to methanol and are inhibited (Xie and Huang 2018). However, certain bacteria can tolerate high concentrations of methanol by altering the structure of fatty acids present in their cell wall (Brännström et al. 2018). The growth of *B. subtilis* strain Q4 was monitored using optical density measurements. *B. subtilis* strain Q4 showed high growth at 5% methanol at 24, 48, and 72 h and showed high growth at 10% methanol at all the incubation hours. However, low growth was observed at 15% methanol concentration at 24, 48, and 72 h at 37 °C. Other study results obtained for the lipase enzyme activity of microbes also showed that, there was a sharp reduction at higher concentrations of methanol (Najjar et al. 2021). The current results depicted that although the growth was reduced at higher concentrations of methanol, strain Q4 was not completely inhibited or killed by methanol. This shows that this strain can be effectively employed for biodiesel production.

During this study, biodiesel production via a *B. subtilis* Q4 MZ841642-mediated trans-esterification of *Chlorella* sp. S5 oil was performed. Other studies also reported that, biodiesel production can be performed through trans-esterification of algal oil (Javed et al. 2018). The production of biodiesel production from algal oil was confirmed by running FTIR. The infrared spectrum of biodiesel usually contains peaks in the frequency (cm^−1^) range of 1735–1750. The FTIR analysis showed a peak at 1735.17 cm^−1^, which confirmed the production of biodiesel. The results of this study agreed with the findings of Najjar et al. (Najjar et al. 2021) who reported produced biodiesel has showed an absorption band at 1743 cm^−1^ by (FTIR).

The GC–MS chromatogram showed that the total FAME content of biodiesel produced was 98.2%, which was in line with quality criteria as specified by both EU14103 (FAMEs > 90%) and American Society for Testing and Materials (ASTM) (FAMEs ≥ 98%). The GC–MS result of the current study was higher than the findings of Najjar et al. (Najjar et al. 2021) who reported obtained GC–MS amount of biodiesel from cooking oil was 83.08%. Similarly, it was also higher than the results reported by Al-Humairi et al. (Al-Humairi et al. 2022) who obtained the maximum biodiesel yield (96 ± 0.2%). In the current study, the FAME content of algal biodiesel was methyl palmitate (32.1%), methyl heptadecanoate (11.2%), methyl myristate (8.2%), methyl lineolate (29.8%), methyl stearate (6.2%), methyl 10-heptadecanoate (7.7%) and methyl hexanoate (3%). Other research finding also supported that, high level of FAME was important for high quality of biodiesel production from Microalgae (Song et al. 2013).

The substrates including lipids, lipid-extracted biomass, starch (positive control), and inoculum (negative control) were used in batch anaerobic digestion mode for 50 days. Figure 8A and B shows that biogas and biomethane yield obtained from lipid-extracted biomass was 580 NmL/g VS and 364.34 NmL/g VS, respectively. Biogas and biomethane yield obtained from whole biomass were 580 NmL/g VS_added_ and 330.34 NmL/g VS_added_, respectively. The results showed that not much difference in the yield of biogas and biomethane exists for whole biomass and lipid extracted biomass. This shows that a process that utilizes oil first for biodiesel production and then lipid extracted biomass for biogas production is more feasible in terms of energy output as it produces two types of biofuels rather than one while having the same yield for biogas. Biomethane yield of the current study was higher than that of reported results of Wu et al. (Wu et al. 2020) who reported the higher 194.63 mL/g VS_added_, and the lowest 104.42 mL/g VS_added_ was obtained.

## Conclusion

The findings of this study conclude that Chlorella sp. S5 can be used effectively for sequential agricultural runoff treatment and resultant biomass can efficiently be used for biodiesel production from algal lipids and biogas from lipid extracted biomass. *B. subtilis* Q4 MZ841642 has mediated trans-esterification of Chlorella sp. S5 oil for better production of biodiesel. Bio-catalytic production of biodiesel confers the advantage of low reaction temperature which cuts down the production cost and non-production of alkali/acid waste which is the byproduct of chemical methods of biodiesel production. Furthermore, utilization of wastewater for algal biomass production provides two advantages: bioremediation and cost-effective biomass production which in turn used for biofuel generation. Hence, the study remains successful in the development of integrated wastewater biorefinery using microalgae at a lab-scale. Therefore, this study has showed that there is high promise role of Chlorella sp. S5 in bioremediation activities of wastewater while producing high yield of biogas and biomethane by using *B. subtilis* Q4 as mediator of trans-esterification of algal oil if it is done at large-scale.

## Data Availability

The data that support the findings of this study are available from the corresponding author upon reasonable request.
